# Comparative study of meningitis dynamics across nine African countries: a global perspective

**DOI:** 10.1186/1476-072X-6-29

**Published:** 2007-07-10

**Authors:** Hélène Broutin, Solenne Philippon, Guillaume Constantin de Magny, Marie-Françoise Courel, Benjamin Sultan, Jean-François Guégan

**Affiliations:** 1 G.E.M.I, UMR CNRS/IRD 2724, Equipe «Evolution des Systèmes Symbiotiques» Institut de Recherche pour le Développement (IRD), 911 Avenue Agropolis, BP 64501, 34394 Montpellier Cedex 5, France; 2 ProdiG, UMR 8586 CNRS/Université. Paris1/Université Paris4/université Paris7/Ecole Pratique des Hautes Etudes (EPHE), 2 rue Valette, 75005 Paris, France; 3 University of Maryland, Institute for Advanced Computer Studies. # 296 Biomolecular Sciences Building. College Park, MD, 20742 USA; 4 UR086 LOCEAN, UMR 7617 IRD-CNRS-UPMC (Université Pierre et Marie Curie), 4 place Jussieu, 75252 Paris cedex 05, France

## Abstract

**Background:**

Meningococcal meningitis (MM) represents an important public health problem especially in the "meningitis belt" in Africa. Although seasonality of epidemics is well known with outbreaks usually starting in the dry season, pluri-annual cycles are still less understood and even studied. In this context, we aimed at study MM cases time series across 9 sahelo-sudanian countries to detect pluri-annual periodicity and determine or not synchrony between dynamics. This global and comparative approach allows a better understanding of MM evolution in time and space in the long-term.

**Results:**

We used the most adapted mathematical tool to time series analyses, the wavelet method. We showed that, despite a strong consensus on the existence of a global pluri-annual cycle of MM epidemics, it is not the case. Indeed, even if a clear cycle is detected in all countries, these cycles are not as permanent and regular as generally admitted since many years. Moreover, no global synchrony was detected although many countries seemed correlated.

**Conclusion:**

These results of the first large-scale study of MM dynamics highlight the strong interest and the necessity of a global survey of MM in order to be able to predict and prevent large epidemics by adapted vaccination strategy. International cooperation in Public Health and cross-disciplines studies are highly recommended to hope controlling this infectious disease.

## Background

Recent insights into epidemiology of infectious diseases have clearly shown the interest of associating empirical with theoretical studies in order to better describe and understand the mechanisms of disease transmission and epidemics emergence, therefore enabling better control of their impact on human health [1-5]. An important evolution in this field, directly coming from ecology, is to develop the comparative analysis of different data sets to obtain a whole picture of disease behaviour in both time and space. The global idea is thus to compare longterm series of disease cases across localities and countries so as to characterize the evolution of epidemics periodicity and to determine the existence of synchrony between time series. Such an approach should considerably improve our understanding of trends in global disease dynamics and thus facilitate the emergence of predictive and quantitative tools for vaccination programs [6]. We aimed at performing such a first comparative study for one of the most important vaccine preventable infectious disease, the Meningococcal meningitis (MM), in 9 African countries of the "meningitis belt": Mali, Burkina Faso, Ghana, Togo, Benin, Niger, Nigeria, Chad and Sudan.

MM is an infectious disease due to the bacteria *Neisseria meningitis *and appears around 200 years in Africa (Egypt, Sudan) and thus spread in West Africa mainly by pilgrims migrations [7]. *Neisseria meningitis *is highly contagious and a person-to-person aerial transmission occurs through respiratory and throat secretions. Epidemics of meningitis occur worldwide but the "meningitis belt" of the Africa Sahel region, which extends from Mali and Côte d'Ivoire in the west to Sudan and Ethiopia in the east, has the greatest incidences of cases, with large epidemics and high mortality rates. Different serotypes of *N. meningitis *are well known as serotypes A (the dominant), C, Y and W135. Epidemics occur throughout Africa in the dry season, coincide with periods of very low humidity and dusty conditions, and disappear with the onset of the rains [8,9]. Climatic conditions, social interactions (e.g. pilgrims), transmission of more virulent serotypes (e.g. recent spread of the W135 serotype in Africa), and susceptibility of populations constitute the major favourable drivers of resurgence and dispersion of the disease.

More than seasonality, it is generally admitted that meningitis epidemics occur every 5 to 10 years in many countries, but this periodicity, to our acknowledge, has not been clearly, statistically, demonstrated in literature. Nowadays, the most adapted mathematical tool applied in epidemiology for the study of long term time series is wavelets analysis [6,10-12]. This tool allows the detection of periodicity as the other classical tools, such as Fourier Transform, but also allows the description of its evolution in time [13].

Thus, we sought to carry out a first comparative analysis using a large set of time series data, and wavelets analysis, for MM in order to (i) detect and quantify global MM dynamics across 9 countries of the "meningitis belt" (periodicity and synchrony), and (ii) show the existence, or not, of a general trend in disease dynamics, focusing on pluri-annual cycles. Finally, we discussed the importance of such large-scale studies to access to a global control of this infection thanks to vaccination.

## Results

### Wavelets analysis, periodicity of meningitis

**Figure 1:**
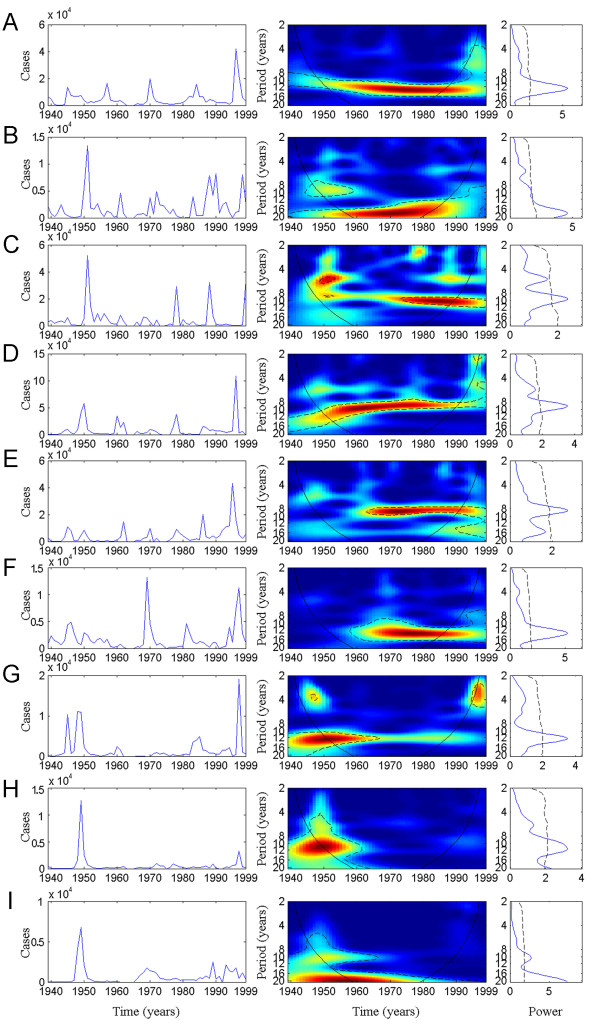
Wavelet analyses of meningitis time series with annual data from 1939 to 1999 across 9 African countries: (A) Burkina Faso, (B) Chad, (C) Sudan; (D) Nigeria, (E) Niger, (F) Mali, (G) Ghana, (H) Togo, (I) Benin. For each country (i) left panel represents the time series of cases in the country (ii) middle panel represents the wavelet power spectrum of meningitis cases (detrended); colours code for increasing spectrum intensity, from blue to red; dotted lines show statistically significant area (threshold of 5% confidence interval); the black curve delimits the cone of influence (region not influenced by edge effects) (iii) right panel corresponds to the mean spectrum (in blue) with its significant threshold value of 5% (black line).

Results are all described on Figure 1. The mean spectra (Figure 1, right graphs) show clear periodicity for all countries. However, wavelets analyses across the 9 time series for the 1939–1999 period show different evolution of meningitis dynamics (Figure 1, middle panels). We can group the different countries in two main domains.

The first group corresponds to countries in which a pluri-annual cycle is detected during the whole time period (1939–1999). In this group, Burkina Faso (BF) (Figure 1-A) shows a quiet constant cycle of around 12 years. In Chad, meningitis seems to present a 9-years cycle before 1960 and then the cycle is reinforced by a 18-years cycle during the whole period of study (Figure 1-B). Meningitis in Sudan shows a periodicity increasing from around 9 years to 12–13 years since the 70's (Figure 1-C). In contrast, periodicity of meningitis seems to decrease in Nigeria from around 12 years to 8–9 years since the mid of the 70's (Figure 1-D).

The second group represents countries in which the periodicity is transient, i.e. not detected before the 60 years or detected till 70's. First, a periodicity of meningitis of 8–10 years is detected in Niger only since the mid of the 60's (Figure 1-E). Similarly, meningitis in Mali shows a clear 12–14-years cycle since the 60's (Figure 1-F). No periodicity was detected in both Niger and Mali between 1939 and 1960. In contrast, Ghana, Togo and Benin all present a clear 12-years cycle till 60's, with no periodicity detected between 1970 and 1999 (Figure 1-G,H,I). Benin shows a 20-years cycle but this result needs high precaution because the analyse is based on a 60 years time series, which is quiet short to detect the existence of a 20-years cycle (only 3 repetitions of the cycle). That is exactly the reason why we preferred not to take this result into further consideration.

Globally, meningitis presents a pluri-annual periodicity in each country under study, even if these 8 to 12 years cycles are not similar and permanent in time.

### Synchrony between countries

**Figure 2:**
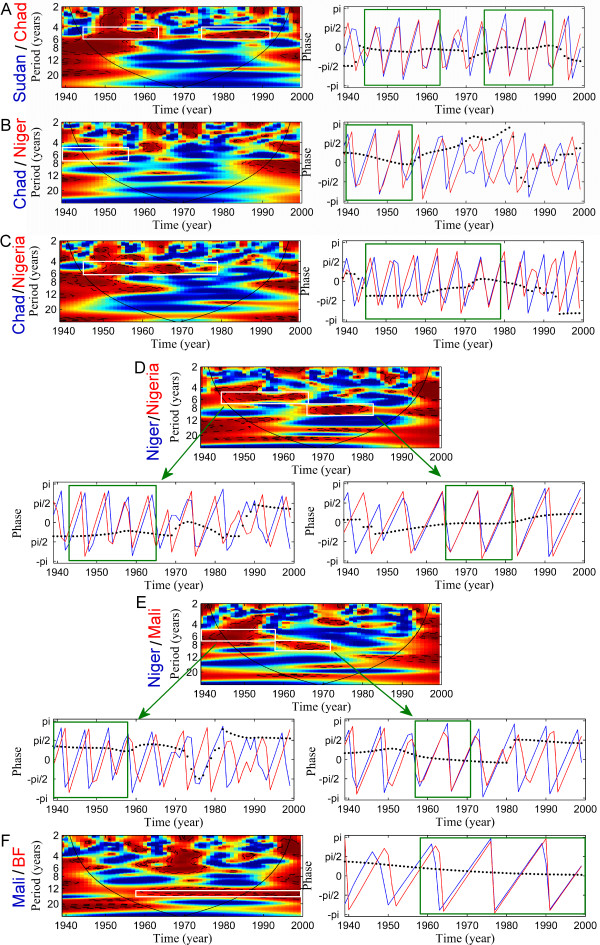
Wavelet coherence and phase analyses of meningitis time series between countries. The left or top panel represents the wavelet coherence (x-axis: year, y-axis: period, in years). Blue, low coherence; red, high coherence. The dotted lines show the α = 5% and α = 10% significance levels based on 500 bootstrapped series. The cone of influence (black curve) indicates the region not influenced by edge effects. The right or bottom panels represent the phase analyse between two countries (in blue and red), based on wavelets for a given periodic band (white band). Green boxes represents the period of time where coherency is significant, i.e. when the interpretation of analyse is possible. Blue lines: first country (name in blue); red lines: second country (name in red); dashed lines: time delay between the two oscillating components (ΔT).

**Figure 3:**
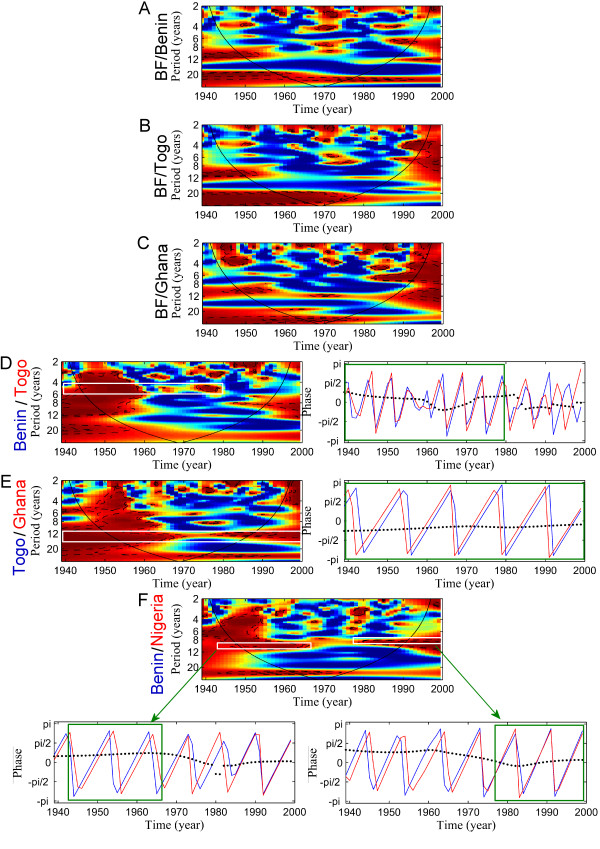
Wavelet coherence and phase analyses of meningitis time series between countries. The left or top panel represents the wavelet coherence (x-axis: year, y-axis: period, in years). Blue, low coherence; red, high coherence. The dotted lines show the α = 5% and α = 10% significance levels based on 500 bootstrapped series. The cone of influence (black curve) indicates the region not influenced by edge effects. The right or bottom panels represent the phase analyse between two countries (in blue and red), based on wavelets for a given periodic band (white band). Green boxes represents the period of time where coherency is significant, i.e. when the interpretation of analyse is possible. Blue lines: first country (name in blue); red lines: second country (name in red); dashed lines: time delay between the two oscillating components (ΔT).

When periodicities are detected for each country, we aim at comparing meningitis dynamics between all the countries. Results are shown on Figures 2 and 3. First, meningitis dynamics in Sudan and Chad are highly coherent and synchronous during the 60 years of study excepted between 1965 and 1975 (Figure 2-A). Pluri-annual epidemics seems to be synchronous between Chad and Niger till 1960 but the coherency is low (Figure 2-B). In contrast, Chad and Niger show coherent meningitis dynamics till the end of the 70's, but Nigeria is 1-year in advance with Chad. A synchrony seems to appear in the 70's but the analyse is not interpretable in this period of time (Figure 2-C). Nigeria and Niger are highly coherent from 1939 to the beginning of the 80's. Nigeria is first 1-year in advance with Niger till the end of the 60's and then both dynamics become synchronous (Figure 2-D).

All these results show that meningitis dynamics are out of phase in these 4 countries but that pluri-annual epidemics become more synchronous since the end of the 60's and the beginning of the 70's. This tendency is also put in light between Niger and Mali, Niger being 1-year in advance with Mali till the end of the 50's and then dynamics are synchronous during the 60's and most part of the 70's (Figure 2-E).

**Figure 4:**
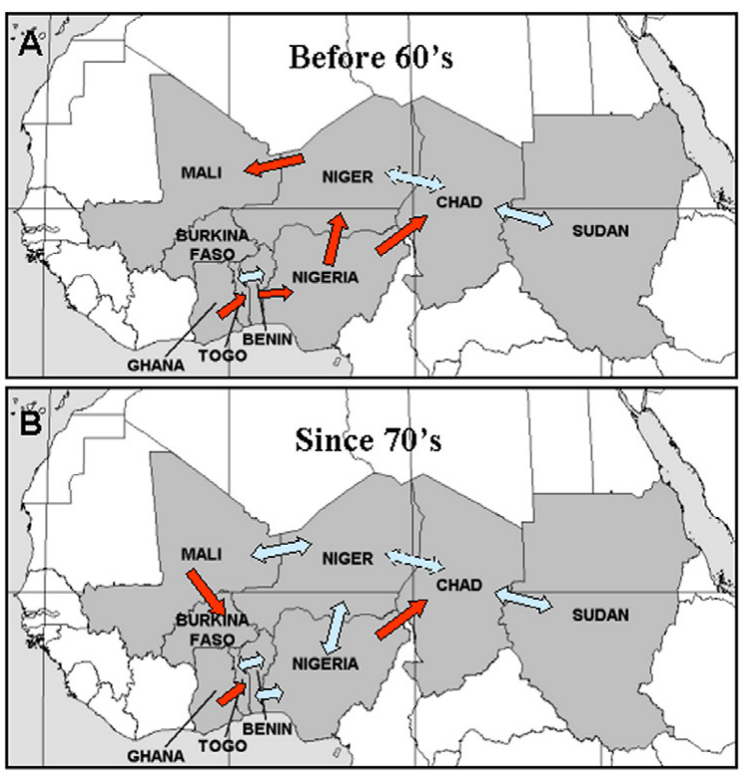
Schematic illustration of the synchrony results obtained (A) before 60's and (B) after 70's for the 9 African countries under study. Each arrow corresponds to significant coherency between two countries: red arrows show the sense of disease spread and blue arrows symbolize synchrony between the 2 countries.

Mali and Burkina Faso (BF) are coherent until 1960 and afterwards Mali was 1-year in advance with BF (Figure 2-F). In order to try to detect this trend "North-South", we compared BF with Benin, Togo and Ghana respectively (Figure 3-A to C). Disease dynamics in Benin, Togo and Ghana are not coherent with meningitis dynamics observed for BF. However, coherency is detected between those countries. Benin and Togo are globally coherent and synchronous till 1980 (Figure 3-D) whereas Ghana is 1-year in advance with Togo during the 60 years (Figure 3-E). Finally, Benin is 1-year in advance with Nigeria from 1939 to the end of the 60's, and as already described above, both countries became synchronous (Figure 3-F). A synthesis of these results is illustrated in Figure 4.

## Discussion

In this study, a MM pluri-annual periodicity is statistically demonstrated in all countries. This periodicity is estimated to be ranged between 8 and 12 years depending of country and the time period. We have to keep in mind that this work only focuses on pluri-annual cycles; unfortunately we did not have access to the monthly data which would have allowed the study of seasonality. Some of the countries show a periodicity throughout the 60 years but, for most of them, periodicity is not detected during the whole time. One particularly interesting result concerns Nigeria: here the cycle is decreasing, showing that large epidemics have been occurring more and more often since the 80's. In fact, this first large-scale comparative study of MM dynamics shows that the pluri-annual periodicity of meningitis is not as evident and regular as it is accepted nowadays. This issue is very important in terms of global control and highlights the importance of such long-term and large-scale studies.

Our study also aimed at comparing the MM dynamics between the different countries in order to detect the existence of synchrony in pluri-annual epidemics. We found a high correlation in MM dynamics between the countries, even if these correlations are not permanent in time. Perfect synchrony or out-of phase synchrony is indeed observed for the majority of comparisons. There is, however, no global synchrony between the 9 countries.

The heterogeneity in meningitis periodicity and synchrony highlights the importance of such comparative studies and surveys for the epidemiological control of global diseases. Indeed, it is easier to control a disease (through e.g. vaccination) if epidemics across different populations are regular and synchronous in time (e.g. if all populations suffer an epidemic peak simultaneously). In contrast, if epidemics are irregular or desynchronised, then disease extinction in one region will probably be temporary, since disease can be reintroduced from neighbouring regions. This reasoning, issued directly from population biology concepts [14,15], reveals an high recommendation of such comparative and synchrony studied in epidemiology in order to attain a global control of infectious diseases in increasingly complex systems [16,17].

Modifications in meningitis periodicities and in both the coherency and the synchrony between dynamics are mainly observed during the 60's and the 70's. These 20 years appear in fact as a period of transition and high perturbation. Synchronization is higher after this period than before it (Figure 4). The first factor which could explain this observation is the start of vaccinations. Vaccination started globally at the end of 1970's in all of these countries [18], but in different proportions and with different types of vaccines. The main difficulty for studying the impact of vaccination is first to know exactly the extent of the vaccine coverage because vaccination programs usually start when an epidemic occurs in the country. Mass vaccination was also used but not for a long time.

Another factor that may be important in the pluri-annual pattern of meningitis epidemics is the impact of climate as for many others climate-sensitive diseases [19]. Harmattan winds, in particular, have a clear effect on meningitis seasonality inducing MM epidemics starting [20]. Their role in the disease's pluri-annual pattern, and the combination between this and other factors such as loss of immunity or the spread of different strains, should now also be explored. This last point is indeed not well documented since major papers about MM concern annual pattern explanation and/or description. It could be useful to focus on these pluri-annual cycles in the "meningitis belt" and to carry out large-scale epidemiological studies such as the one presented here.

Obviously, the main question is how to better control MM. There is an ongoing debate about the best vaccination strategy to adopt: reactive vaccination, selective vaccination or mass vaccination [21-24]. The main strategy used today consists in a reactive vaccination which means that vaccination starts in a population once an alert threshold of incidence (e.g. 10/100000 persons) is reached. The main limitation of this strategy is that vaccination starts after the beginning of the epidemics, decreasing the efficiency of the global population protection. Nowadays, the main objective is to better understand emergence and spread mechanisms of MM in order to be able to predict epidemics *before *the first human case appear. For this purpose international cooperation during a meningitis epidemic is crucial [21]. An international system of alert, which could combined real time climate survey and real time MM incidence threshold approach should indeed help to react quickly and trigger an effective mass vaccination in the countries concerned.

This work is the first large-scale description of MM dynamics. The next step now is to perform new analysis of MM dynamics comparisons using more detailed epidemiological data (e.g. monthly data, by regions...) as already done at a local scale in Northern Togo [25]. The inclusion of vaccination and the genetic variation of serotypes data is also requested for next studies as well as long-term climatic factors.

## Conclusion

In conclusion, large-scale studies such as the ones presented in this paper are essential to understanding global disease dynamics. Nevertheless, these studies need to be completed with local information in order to have access to the whole picture of the disease evolution in time and space. In addition, as climate is clearly related to MM epidemics, this factor should be studied at global scale between countries of the "meningitis belt" to try to detect general patterns and, even, to be able to predict epidemics via climate survey. This issue emphasizes the importance of cross-disciplines studies to better understand MM evolution and dynamics. Finally, an eye should be kept on countries around the meningitis belt as MM is spreading beyond its perimeter as exemplified by the case of Cameroon [26,27].

## Methods

### Epidemiological Data

This work is based on data extracted from the WHO website and from Lapeyssonie [18]. They correspond to annual notifications of meningitis from 1939 and 1999 in 9 countries in the sub-Saharan area: Sudan, Chad, Niger, Nigeria, Mali, Burkina Faso (BF), Ghana, Togo and Benin. Annual data allowed us to interest only to pluri-annual cycles and no results will be obtain concerning seasonality. Heterogeneity in data due to various diagnosis and survey methods could imply underreporting in this database but we aimed at describe the global tendency of MM dynamics so that this heterogeneity could not affect our results. Moreover, we used wavelet analyses which is more a qualitative methodology rather than quantitative. Three years were not documented, i.e. 1963–1964–1965. We then decided to perform first analyses of time-series between 1966 and 1999, and we realised the same procedure but for the over all period 1939–1999. We obtained the same results, and thus we decided to use the whole data base maintaining the years with 0 case because this lack didn't interfere in the results. The unique constraint was to interpret with precaution periodicity of 3 or 4 years in this period time.

### Wavelet analysis – periodicity

In order to detect periodicity in time series, we used wavelet analysis [10,11] a well suited tool to explore local variations in frequency (and periodicity) as time progresses and the evolution through time of these periodic components [10]. In comparison with other classical tools as spectral analysis and correlograms, that means we can detect changes in periodicity in time. It is particularly well suited to our study, as it allowed us to determine whether periodic components of our series change in time and compare for each period all the countries. We used the Morlet wavelet and all analyses were performed with Matlab 6.5 software. For more technical details on this tool, see [6,13].

### Coherency and phase analyses – synchrony

Coherence is similar to some classical correlation but for the oscillating components in a given frequency mode. Wavelet coherency generalizes the possibilities of wavelets for quantifying the dependencies between two signals. These tools allow us to quantify the synchrony of two time series in a given periodic mode, *i.e. *to quantify if two time series tend to oscillate simultaneously, rising and falling together with the same period. In complement to wavelet analysis, we can use phase analysis [28] to characterize the association between signals [11], and also compute the instantaneous time lag between the time series [6]. This analyse is pertinent only for band of periods where both time series are coherent, making it dependent of the coherency analyses.

## Competing interests

The author(s) declare that they have no competing interests.

## Authors' contributions

HB conceived of the study, carried out all analyses and wrote the draft of manuscript. SP collected data and helped to draft manuscript. GCdM participated in the design of the study and helped to wavelets analyses. MFC and BS helped to draft the manuscript. JFG conceived of the study and participated to draft the manuscript. All authors read and approved the final manuscript.
